# Molecular markers of reduced behavioral sensitivity to transfluthrin in *Anopheles gambiae s.s.* from Western Kenya

**DOI:** 10.1186/s12864-025-11755-y

**Published:** 2025-06-05

**Authors:** Stephen Okeyo, Dieunel Derilus, Lucy Mackenzie Impoinvil, Nsa Dada, Diana Omoke, Helga Saizonou, Cynthia Awuor Odhiambo, Nicola Mulder, Gerald Juma, Benard W. Kulohoma, John E. Gimnig, Luc S. Djogbénou, Audrey Lenhart, Eric Ochomo

**Affiliations:** 1 Kenya Medical Research Institute (KEMRI), Centre for Global Health Research (CGHR), Kisumu, Kenya; 2 Department of Biochemistry, University of Nairobi, Nairobi, Kenya; 3 Division of Parasitic Diseases and Malaria, Entomology Branch, National Center for Emerging and Zoonotic Infectious Diseases, Centers for Disease Control and Prevention, 1600 Clifton Rd, Atlanta, GA 30329 USA; 4 Tropical Infectious Diseases Research Centre (TIDRC), University of Abomey-Calavi (UAC), Abomey-Calavi, Benin; 5 School of Life Sciences, Arizona State University, Tempe, AZ USA; 6 Human, Heredity, and Health in Africa H3A Bionet network, University of Cape Town ZA, Cape Town, South Africa; 7 IAVI Africa, Nairobi, Kenya; 8 Regional Institute of Public Health (IRSP), Ouidah, Benin; 9 Department of Vector Biology, Liverpool School of Tropical Medicine, Liverpool, United Kingdom

**Keywords:** *An. gambiae s.s*, Transfluthrin, Insecticide resistance, Spatial repellents, RNA-seq, CYP12 F12, *kdr* mutation

## Abstract

**Background:**

The emergence and spread of insecticide resistance in malaria vectors threatens vector control efforts. The use of spatial repellent products (SR) containing volatile insecticides such as transfluthrin offer a promising complementary strategy to current vector control tools. Here, we employed whole transcriptome analysis to investigate the molecular mechanisms underlying reduced behavioral sensitivity to transfluthrin in two pyrethroid-resistant populations of *Anopheles gambiae s.s.* Using a high-throughput screening system (HITSS), we evaluated 600 mosquitoes across three populations (Bungoma field population, the insecticide-resistant Pimperena lab strain, and the susceptible Kisumu lab strain), categorizing them as responders or non-responders based on their SR avoidance behavior. Non-responders exhibited significantly reduced repellency (spatial activity index < 0.1) at standard transfluthrin concentrations (0.0025 μg/ml).

**Results:**

RNA sequencing of pooled samples (*n* = 10 mosquitoes per pool, three replicates per condition) revealed distinct transcriptional profiles between responders and non-responders. The cytochrome P450 gene CYP12F12 showed significant overexpression (FC = 36.6389, *p* < 0.001) in Bungoma non-responders, suggesting its potential role in transfluthrin metabolism. Additionally, we observed population-specific distributions of voltage-gated sodium channel mutations, with fixation of kdr L995F in Pimperena non-responders and elevated frequency (80–100%) of *kdr* L995S in Bungoma non-responders.

**Conclusions:**

These findings provide the first molecular evidence linking both metabolic and target-site mechanisms to reduced behavioral sensitivity to transfluthrin in malaria vectors. The co-occurrence of CYP12F12 overexpression and *kdr* mutations suggests multiple resistance mechanisms may affect spatial repellent efficacy, highlighting the need for resistance monitoring in spatial repellent deployment strategies.

**Graphical Abstract:**

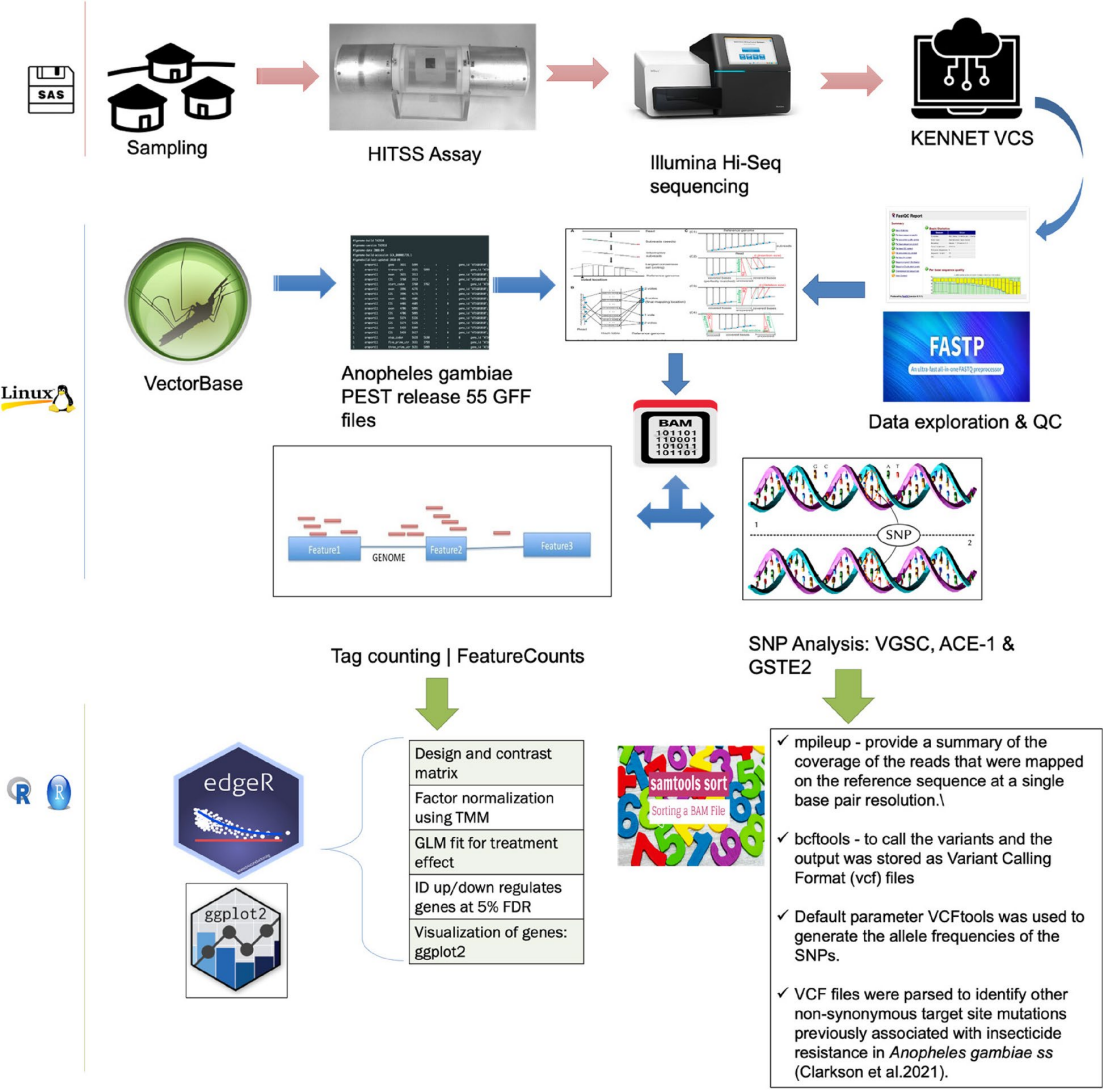

**Supplementary Information:**

The online version contains supplementary material available at 10.1186/s12864-025-11755-y.

## Background

Malaria remains a significant public health burden, with an estimated 263 million cases and 597,000 deaths reported globally in 2023 [1]. The primary tools for vector control continue to be insecticide treated nets (ITNs) and indoor residual spraying (IRS) [2]. However, the widespread emergence of insecticide resistance, including behavioural resistance where mosquitoes evolve to avoid interventions, threatens the effectiveness of these interventions [2, 3].

Several promising complementary strategies are currently under evaluation, including use of attractive targeted sugar baits (ATSBs) [4, 5], house modifications [6–8], administration of endectocides [9], gene-drive technologies [10], and spatial repellents (SRs) [11, 12]. Spatial repellents offer unique advantages for malaria control by creating protective spaces that deter mosquito entry [13, 14], reducing human-vector contact without requiring direct mosquito contact, and providing protection in settings where traditional interventions face limitations. In addition to complementing traditional interventions such as ITNs, SRs may be particularly valuable in scenarios where these tools face challenges, including areas with outdoor or early evening biting vectors, regions with inconsistent ITN usage, emergency situations (e.g., flooding, refugee camps) and settings where IRS implementation is impractical [15].

Transfluthrin represents a unique pyrethroid structure characterized by a polyfluorinated benzyl ring, distinguishing it from conventional pyrethroids used in ITNs. This structural difference, particularly the absence of the classical 3-phenoxybenzyl moiety found in traditional pyrethroids, suggests potentially distinct interactions with known resistance mechanisms [14, 16, 17]. While traditional pyrethroids like permethrin and deltamethrin primarily act through direct contact, transfluthrin's high vapor pressure (1.5 × 10–3 Pa at 20 °C) enables spatial repellency through airborne distribution.

While transfluthrin-based SRs hold considerable promise, their long-term use and effectiveness hinges on the understanding of how pyrethroid-resistant mosquitoes respond to transfluthrin. Despite transfluthrin's unique chemical structure compared to conventional pyrethroids used in vector control, we anticipated the potential for cross resistance where established target site and metabolic mechanisms active against type I&II pyrethroids would be activated in response to transfluthrin exposure. For this reason, we selected pyrethroid-resistant strains to investigate potential markers [18].

Using a combination of behavioural assays and whole-transcriptome analysis, we investigated the molecular mechanisms underlying reduced behavioural sensitivity to transfluthrin in *An. gambiae s.s*. Differential gene expression associated with behavioral insensitivity to transfluthrin was determined through whole-transcriptome analyses of individuals assayed for repellency behaviors using a High Throughput Screening System (HITSS) [19]. By elucidating these mechanisms, we contribute the first molecular correlates of behavioral resistance and provide valuable insights to resistance management strategies that incorporate spatial repellents.

## Methodology

### Mosquito populations

Three *An. gambiae* ss. mosquito populations were used for this experiment. Two were laboratory reference populations: the pyrethroid susceptible *An. gambiae s.s.* Kisumu strain and the pyrethroid resistant *An. gambiae ss* Pimperena strain, both obtained from the MR4 [20]. The third was adult filial 1 (*F*1) generation raised from field collected adult mosquitoes from Bumula sub-county (0° 34′ 14.052''N, 34° 33′ 36.0036''E) in Bungoma county, western Kenya. The collections were conducted between March and October 2017 and verbal consent was obtained from household heads for collections. The mosquitoes were transported to the Entomology laboratory at the Kenya Medical Research Institute in Kisumu, Kenya for oviposition. The progeny was hatched and reared to adult stage, and 3–5 days old non-blood fed F1 females were used to conduct behavioral assays. Laboratory conditions were consistently maintained at temperature 27 ± 2 °C and humidity 70 ± 10% on a 12:12 h light: dark cycle while adults were provided 10% sugar solution ad libitum [21].

### Behavioural repellency assay

Repellency was measured in mosquitoes exposed to transfluthrin-treated netting in a HITSS treatment chamber [19, 22]. Nettings were treated underneath a chemical fume hood, always treating the control netting first followed by treatment netting. After 15 min, dry nettings were attached to inner metal cylinder of corresponding metal test chambers using a magnet. HITSS assays were conducted at the KEMRI laboratory in Kisumu (for the wild Bungoma population), and the CDC Entomology Branch laboratory in Atlanta, USA (for the Pimperena and Kisumu colonies). The HITSS test system was comprised of a clear central cylinder, connected to two metallic cylinders representing the treatment (containing transfluthrin-treated netting) and control (containing non-treated netting) chambers using butterfly valves [19]. For each assay, 20 non-blood fed, female *An. gambiae s.s.* were introduced into the center clear chamber using mechanical aspiration and any mechanical knockdown (KD) due to the transfer process was recorded. The chamber was then covered with a dark cloth to allow the mosquitoes to acclimatise for 10 min. After 10 min, any remaining mechanical KD due to the transfer process recorded, and the butterfly valves of each test chamber were opened simultaneously allowing mosquitoes to access the treatment and control chambers. The clear chamber was covered using a dark cloth during the experiment to control for bias introduced by light. The valves were closed after 10 min and the number of mosquitoes in both metal chambers and the clear central chamber recorded [22, 23]. Mosquitoes that moved to the treatment chamber section were labelled “non responders”, indicating that the presence of transfluthrin did not induce repellency, while those that moved to the control chamber were labelled “responders”; those that remained in the clear chamber were discarded. All the responders, non-responders and a further group of mosquitoes from the same batch that weren’t used in the assays (‘unexposed’) were preserved in RNA-Later and stored at −4 °C before being shipped (within 5 days), to the CDC laboratory where they were stored at −80 °C prior to subsequent molecular assays. After each experiment, the HITSS chamber was washed using acetone starting with the control pan and then the treatment pan, making sure to tilt tray such that acetone washes over clips used to hold netting strips.

### Spatial activity index (SAI)

Spatial activity index is defined as the ratio of mosquitoes that do not enter the treatment chamber in comparison to all mosquitoes moving within the HITSS assay unit and is calculated as follows (Eq. 1):

Equation 1: Calculation of spatial activity index (SAI)

$$SAI=\left[\frac{\left(Nc-Nt\right)}{\left(Nc+Nt\right)}\right]*(\frac{Nm}{N})$$

where:

i) SAI is spatial activity index
ii) Nc is the total number of mosquitoes in the control metal chamber
iii) Nt is the total number of mosquitoes in the treatment metal chamber
iv) Nm is the total number of mosquitoes in both metal chambers
v) N is the total number of mosquitoes in the entire test unit

The SAI value ranges from −1 to 1, with zero indicating no response to stimulus, −1 indicating an attractant response, and 1 indicating a repellent response. If no movement is noted within the HITSS chamber system (i.e., Nt = 0, Nc = 0), the experiment is still valid although the spatial activity index is 0 [19, 22]. The spatial activity index was calculated from each replicate, and the mean index for each transfluthrin dosage was analyzed using probit-plane regression analysis in R v.4.0.0 at 95% confidence intervals. Based on the dose at which mosquitoes from each of the three populations elicited the highest SAI and at the lowest knockdown, mosquitoes were selected for molecular analyses. Knockdown of females was observed within 60 min of CDC bottle assays within the same populations and concentrations as those used in the behavioural assay.

### RNA extraction, RNA-Seq library preparation and sequencing

RNA was extracted from triplicate pools of 10 mosquitoes representing each phenotype (responders, non-responders, unexposed) per population. RNA was extracted using the Applied Biosystems Arcturus PicoPure RNA Isolation kit (Arcturus, Applied Biosystems, USA) according to the manufacturer’s instructions. RNA was quantified using the Agilent RNA ScreenTape 4200 assay, according to the manufacturers’ protocols (Agilent Technologies, Palo Alto, CA, USA). RNA was DNase treated using Baseline-ZERO DNase (Epicentre, Illumina) and ribosomal RNA depleted using Ribo-Zero rRNA removal kit (Human/Mouse/Rat) (Epicentre, Illumina). Library preparation was carried out using the ScriptSeq v2 RNASeq Library Preparation kit according to the manufacturer’s instructions (Epicentre, Illumina). Each library was barcoded, and equal amounts of each library pooled and sequenced (2 × 125 bp paired-end sequencing) on an Illumina HiSeq 2500 sequencer, using v2 chemistry. Sequencing was done at the Biotechnology Core Facility at CDC, Atlanta, USA.

### Data transfer and analysis

Raw sequences were transferred to Kenya using Sharepoint® (One Microsoft Way Redmond, WA, USA). The data were then uploaded onto a virtual compute server (VCS) on which high performance computing was provided and managed by the Kenya Education Network Trust (KENET), Nairobi, Kenya. RNA-Seq analysis pipelines were configured onto this platform with the support of H3 A BioNET, South Africa.

### RNA-Seq data analysis

On the VCS, the raw reads were analyzed for quality using FastQC, v0.11.5 [24]. To explore relative distribution of expressed transcripts levels between samples with markedly significant difference in distribution, Trimmed Mean of the M-values (TMM) was used to estimate normalization factors used directly in the statistical model for differential expression (DE) testing, while preserving sample properties of the data. To evaluate the degree of relatedness between mosquito strains and biological replicates, a Principal Component Analysis (PCA) was performed on the normalized RNA-Seq data. To confirm the overall similarities, differences, and relationships between RNA-Seq samples, a heatmap and hierarchical clustering of the Pearson’s correlation coefficient of the normalized expression profile between all possible pairs of samples was performed. The reads from lanes 1 and 2 of the sequencer flow cells were merged to increase the overall read coverage. The reads were trimmed to remove Illumina adapter sequences and to remove low quality sequences using Fastp, v0.20.1, with a minimum window quality score of 20 [25]. Read pairs where one or both reads were shorter than 25 bp after trimming were removed. The trimmed reads were aligned to the *An. gambiae* PEST reference genome assembly (https://vectorbase.org/vectorbase/app/downloads/release55/AgambiaePEST/) using ‘Subjunc’, v1.6.0, part of the subread aligner package, with default parameters [26]. Alignments were filtered to remove reads with low mapping quality scores (< 10).

Tag counting was done using featurecounts, v1.6.0, part of the sub-read aligner package [26]. Aligned reads that overlapped coding sequence (CDS) features by at least 1 bp in the sense orientation were counted. The tabulated tag counts were used as input for differential gene expression analysis in edge R, a package in R, v4.0.0 [27, 28]. Aligned reads were assigned to genes quantifying the levels of gene expression and these data were used to compare non-responders from the Bungoma (field) population to (i) responder mosquitoes from the same location, (ii) the fully susceptible Kisumu colony (responders) and (iii) to the resistant Pimperena colony (non-responders). To remove the effect of noise and limitedly expressed genes, only genes where at least one sample had a tag count of 50 or more were analyzed.

### Differential gene expression associated with reduced repellency to transfluthrin

Identification of DE genes associated with non-response to transfluthrin treatment was conducted using set parameters as follows: *P* < 0.05 and fold change (FC) > 2 in the comparisons between non-responder, unexposed control, and responder mosquito. A three pairwise comparison was conducted for each population: non-responders vs same population responders (NR-R), non-responders vs unexposed control (NR-C) and non-responders vs Kisumu responders (NR-R). This approach aimed to identify genes associated with non-response to transfluthrin in the Bungoma population. The first comparison (NR-R) identified genes overexpressed in non-responders compared to responders from the same location (accounting for geographical background). The second comparison (NR-C) identified genes overexpressed in non-responders compared to unexposed controls (controlling for background levels of gene expression in the population). Finally, the third comparison non-responders compared to Kisumu responders (NR-KR) identified genes differentially expressed between non-responders and responders from the susceptible Kisumu strain (allowing for the comparison of non-responders with a susceptible reference strain).

### Gene set enrichment analysis

The gene set AgamP4.14(Genome version = GCA_000005575.2) of the *An. gambiae* PEST reference genome was retrieved from VectorBase release 55 and used for gene ontology analysis. This version release contains a total of 13,845 genes, 13,107 of which are protein coding genes with 738 non-protein coding genes and 9 pseudogenes (https://vectorbase.org/vectorbase/app/downloads/release-55/AgambiaePEST/fasta/data/). Gene Set Enrichment Analysis (GSEA) was carried out on DE genes using default parameters in GOATools (https://github.com/tanghaibao/goatools), a python package containing a library to process over- and under-representation of certain gene ontology (GO) terms, based on Fishers’ exact test. In this study, a False Discovery Rate (FDR) adjusted *p*-value of < 0.05 was set to determine the significantly enriched GO terms associated with the list of DEGs [29]. All the enriched GO terms were then summarized using the python visualization package GO-Figure! [30].

### Analysis of non-synonymous single nucleotide polymorphisms (SNPs)

Sorted binary alignment and map (BAM) files obtained from alignments of raw reads against indexed *An. gambiae* PEST reference genome release 55 were used to identify target site mutations. Target site mutations in the voltage-gated sodium channel (VGSC) gene (the target site for pyrethroids and DDT) have been previously described in *An. gambiae ss* [31]. The SAMtools mpileup utility was used to pileup all the BAM files so as to provide a summary of the coverage of the reads that were mapped on the reference sequence at a single base pair resolution. SAMtools bcftools utility was then used for variant calling and the output was stored as Variant Calling Format (vcf) files. Default parameter VCFtools was used to generate the allele frequencies of the SNPs. The aligned reads were also parsed to identify other non-synonymous target site mutations previously associated with insecticide resistance in *An. gambiae ss* found at other loci [31].

## Results

### Spatial activity index of the mosquito populations

Population responses to transfluthrin were evaluated using the HITSS assay across multiple concentrations. The SAI of the mosquito populations at different doses is presented in Table 1. Optimal doses (highlighted) were selected based on maximum SAI with minimal knockdown for subsequent molecular analysis.

**Table 1:**
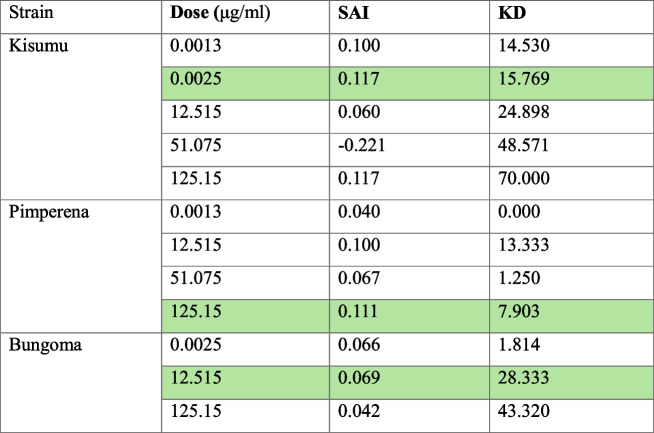
Results of the HITSS assay for the three different mosquito strains (Kisumu, Pimperena and Bungoma) at different transfluthrin treatment doses. For each strain, the highlighted (green) rows show population-specific doses at which the highest SAI was observed with the lowest knock down (KD)

*KD* Knockdown, *SAI* Spatial activity index

### RNA sequencing quality control, data exploration and mapping metrics

RNA sequencing generated high-quality data across all populations. A summary of the quality of the raw reads was obtained for all samples. The data were summarized into a table providing information on the quality and quantity of the sequencing data in Supplementary Material 1. Across the Bungoma, Kisumu, and Pimperena populations, post-filtering read percentages averaged 97.1%, 96.7%, and 96%, respectively. Mapping to the *An. gambiae* PEST reference genome, read percentages averaged 90.7%, 94.3%, and 88.3%, respectively.

**Fig. 1:**
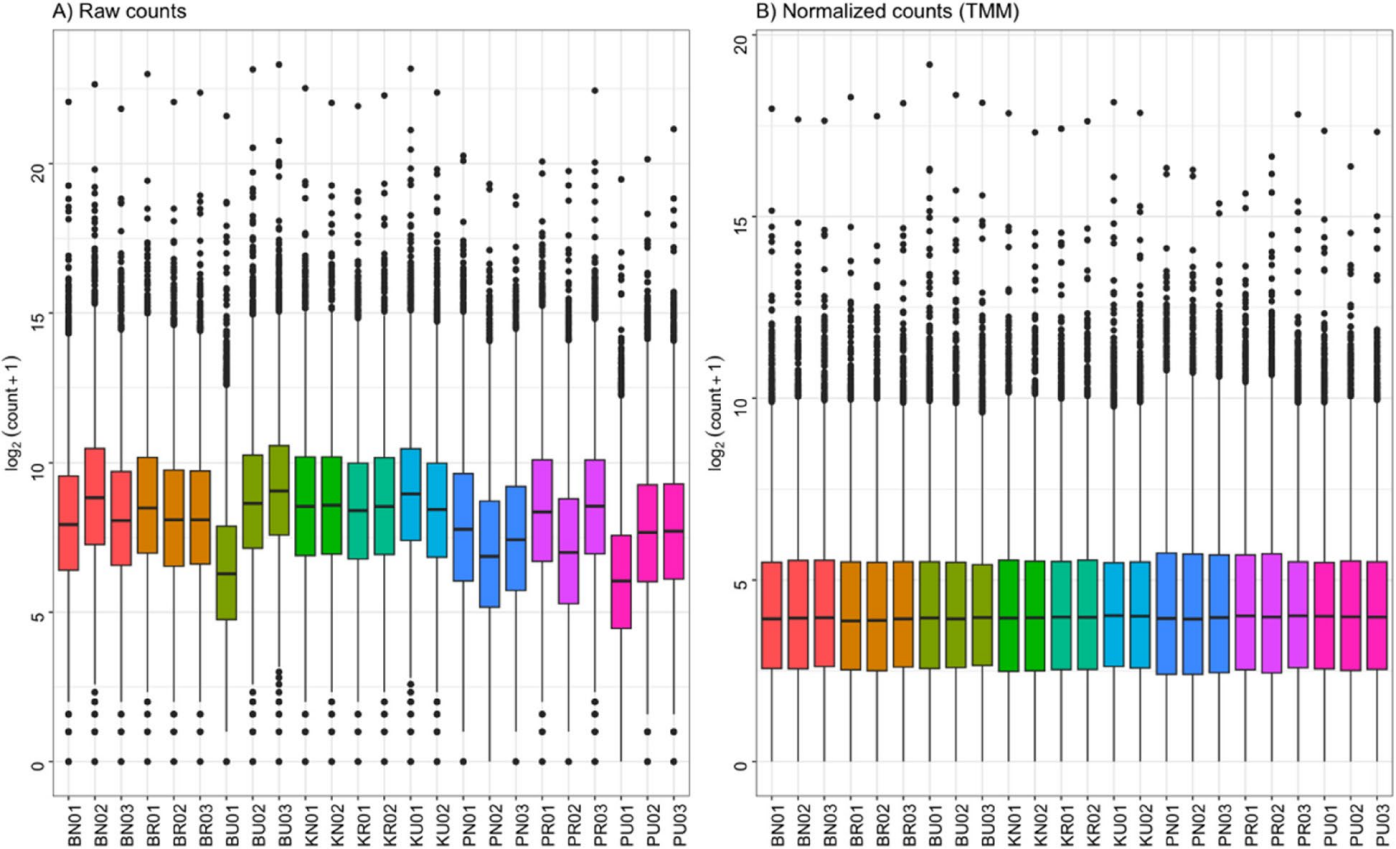
Normalization of RNA-Seq library data. **A** Distribution of raw reads from the sequencing; RNA-Seq libraries are colored based on technical replicates. **B** TMM normalization to resolve technical variation between samples in the experiment. Raw counts were log transformed to further reduce differences in dimensions of counts before the normalization step

Before normalization, the raw read counts had variable mean ratios of counts (Fig. 1). After TMM normalization, the read counts were standardized across all samples and the number of reads in the library were scaled to a mean log value ~ 5 across all sequence libraries (Fig. 1). The pre-normalization mean count ratios exhibited a distribution within the log_2_(4–12) interval. Following TMM normalization, the mean count ratios across all samples from the three populations converged to an average of log_2_(5).

The PCA analysis revealed 66.94% of the total variation could be explained by PC1, while 13.19% could be explained by PC2 (Supplementary Material 2). The clustering pattern of the RNA-Seq library from the hierarchical clustering approach was similar to that observed in the PCA but supported a stronger separation of samples by treatment response (transfluthrin responders vs non-responders) (Supplementary Material 3). From both clustering analysis approaches, the RNA-Seq libraries clustered in part by population geographical origin.

### Analysis of differential gene expression (DE)

**Fig. 2:**
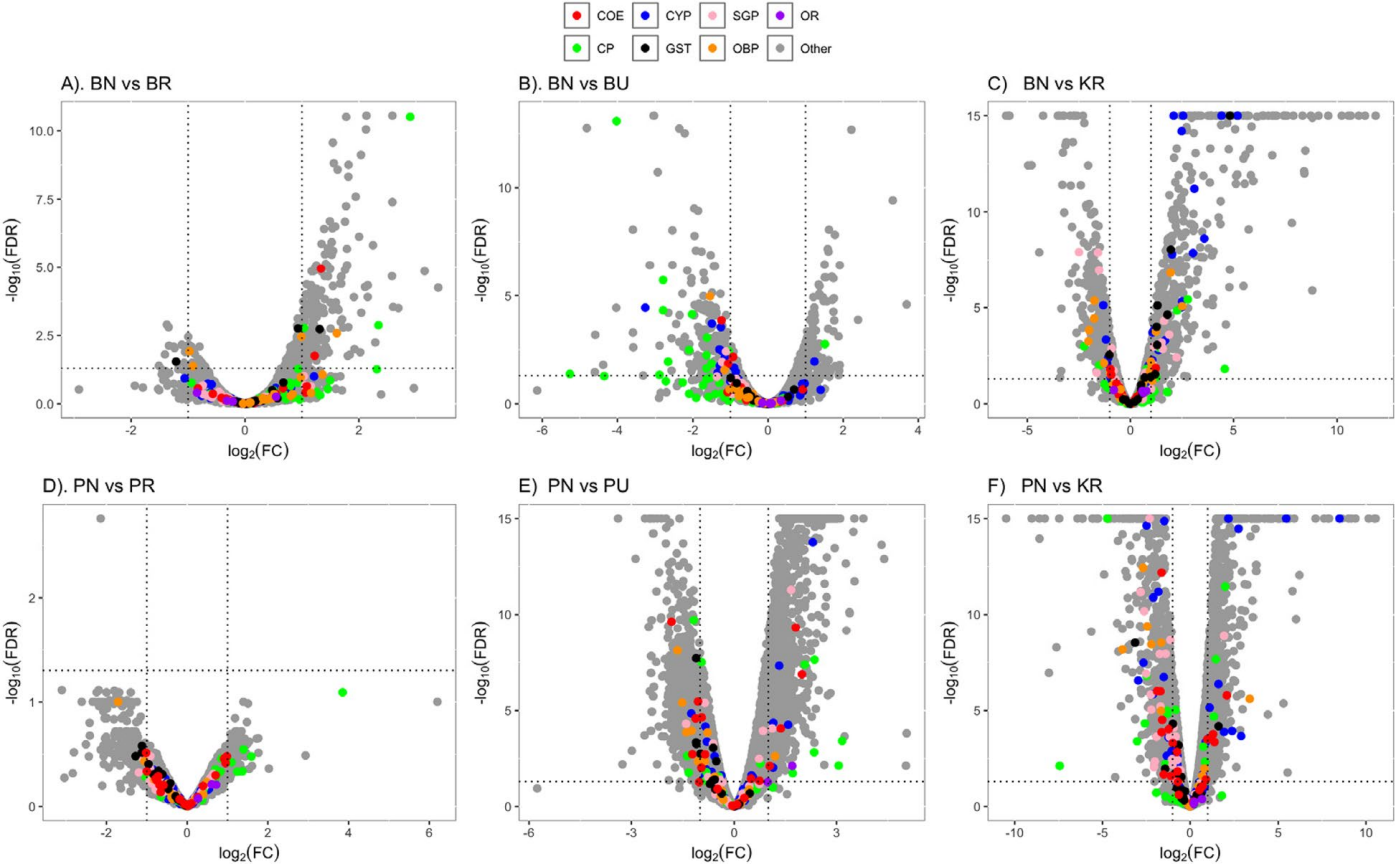
Volcano plots showing gene expression profiles in *An. gambiae s.s.* for the comparisons: (**A**) Bungoma non-responders versus Bungoma responders (BN vs BR); (**B**) Bungoma non-responders versus Bungoma unexposed (BN vs BU); (**C**) Bungoma non-responders versus Kisumu responders (BN-KR); (**D**) Pimperena non-responders versus Pimperena responders (PN vs PR); (**E**) Pimperena non-responders versus Pimperena unexposed (PN vs PU); (**F**) Pimperena non-responders versus Kisumu responders (PN vs KR). Red, green, black, pink, and grey points on volcano plots indicate gene families with major role in metabolic resistance to insecticides: cytochrome P450 monooxygenases (CYP, blue); glutathione-S transferases (GST, black); carboxylesterases (COE, red); cuticular proteins (CP, green); Salivary gland proteins (SGP, pink); Odorant binding proteins (OBP, orange); Odorant receptors (OR, purple). In each plot, genes over-expressed in the population are > 0 on the x-axis while genes under-expressed in the population are < 0 on the x-axis. Vertical dotted line indicates twofold expression differences, and the horizontal dotted line indicates an adjusted *p*-value of 0.05

The results of the differential gene expression analyses comparing the transcriptomic profiles of the different mosquito populations in response to transfluthrin exposure are summarized in Table 2 and visualized in volcano plots Fig. 2. Comparative transcriptomic analysis between Pimperena non-responders and responders revealed a single differentially expressed gene, while a significant overexpression of genes was observed when comparing Pimperena non-responders to Pimperena unexposed. A detailed summary of all comparisons is available in Supplementary Material 4.

**Table 2:** Differential gene expression analysis descriptive summary statistics. DE = differentially expressed, FC = Fold change and adj*P* = *P*-value adjusted for multiple testing by the method of Benjamini and Hochberg [32] BN: Bungoma non-responders; BR: Bungoma responders; BU: Bungoma unexposed; KN: Kisumu non-responders; KU: Kisumu unexposed; PN: Pimperena non-responders; PR: Pimperena responders; PU: Pimperena unexposed. From the HITSS assay, mosquitoes that moved to the treatment chamber section were labelled “non responders”, indicating that the presence of transfluthrin did not induce any repellency, while those that moved to the control chamber were labelled “responders”; those that remained in the clear chamber were discarded thus not part of the analysis

Comparison	No. of genes	DE genes(adj*P* < 0.05)		DE genes(adj*P* < 0.01)		DE genes(adj*P* < 0.05) & (|FC|> 2)		DE genes(adj*P* < 0.01) & (|FC|> 2)	
		Up	Down	Up	Down	Up	Down	Up	Down
PN vs KR	9470	2384	2205	1886	1600	1160	1044	1111	981
PN vs PU	8943	2682	2729	2240	2017	1380	939	1351	897
PN vs PR	9528	0	1	0	1	0	1	0	1
BN vs KR	9748	741	981	637	432	649	408	565	342
BN vs BU	9996	205	263	99	142	111	214	78	136
BN vs BR	9780	283	60	185	18	193	31	159	14

### Comparing Bungoma non-responders to Bungoma and Kisumu responders

The number of genes significantly differentially expressed (DE) for the BN-KR comparison was 1057 (649 up-regulated and 408 down-regulated) and BN-BR 224 (193 up-regulated and 31 down-regulated). The BN-BU comparison had 325 DE genes (111 up-regulated and 214 down-regulated), as shown in Table 2.

In comparing shared DE genes between (BN-KR/BN-BR/BN-BU), 3 genes were commonly shared, 2 up-regulated (AGAP011044, unspecified product; AGAP001124, aminomethyltransferase) and 1 down regulated (AGAP008295 (Trypsin 2)). A summary of fold change (FC) values is available in Supplementary Material 5.

The top 10 detoxification genes with the highest FC between the non-responder vs responder comparisons were a CYP12 F2 (AGAP008020, FC = 36.6389); GSTE2 (AGAP009194, FC = 28.3665); CYP6M2 (AGAP008212, FC = 21.3579);CYP6P4 (AGAP002867, FC = 8.57286949); CPAP3B(AGAP009790,FC = 6.87030903); CYP6P3 (AGAP002865,FC = 5.88653066);CYP6Z2(AGAP008218,FC = 5.6817547);CYP9 K1(AGAP000818,FC = 5.60413546);CYP4 C27(AGAP009246, FC = 5.05599285; D7L1 (AGAP008278, FC = 4.71874157). Insect odorant binding proteins, olfactory genes and odorant receptors had fewer differentially expressed (DE) genes.

### Comparing Pimperena non-responders to Pimperena and Kisumu responders

**Fig. 3:**
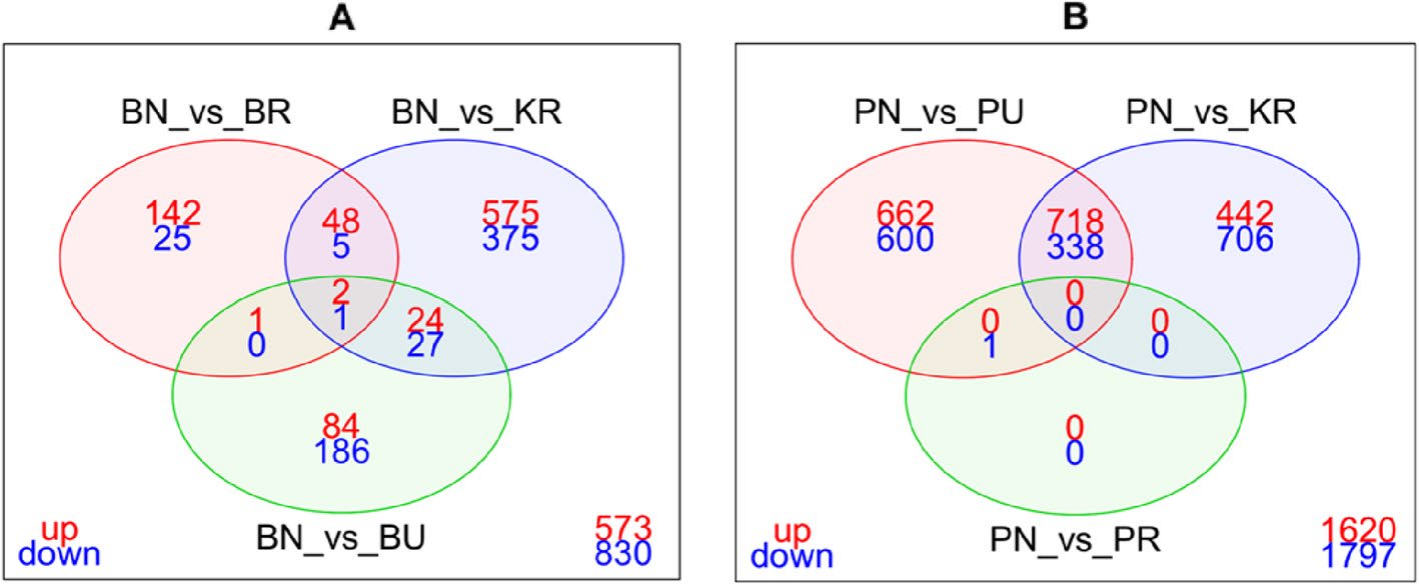
Venn diagrams summarizing the numbers of differentially expressed (DE) genes between non-responders (N), unexposed (U) and responders (R) with a corrected/adjusted *p*-value < 0.01. **A** DE genes in the Bungoma population non-responder samples; **B** DE genes in the Pimperena population non-responder samples

The number of significantly DE genes in the PR-KR comparison was 2204 (1160 up-regulated and 1044 down-regulated) while the PN-PR comparison had 1 DE (0 up-regulated and 1 down-regulated). The PN-PU comparison had 2319 DE (1380 up-regulated and 939 down-regulated) (Fig. 3). There were no shared DE genes among the three comparisons (PR-KR)/(PN-PR)/(PN-PU).

### Comparing Bungoma non-responders against Pimperena non-responders

**Fig. 4:**
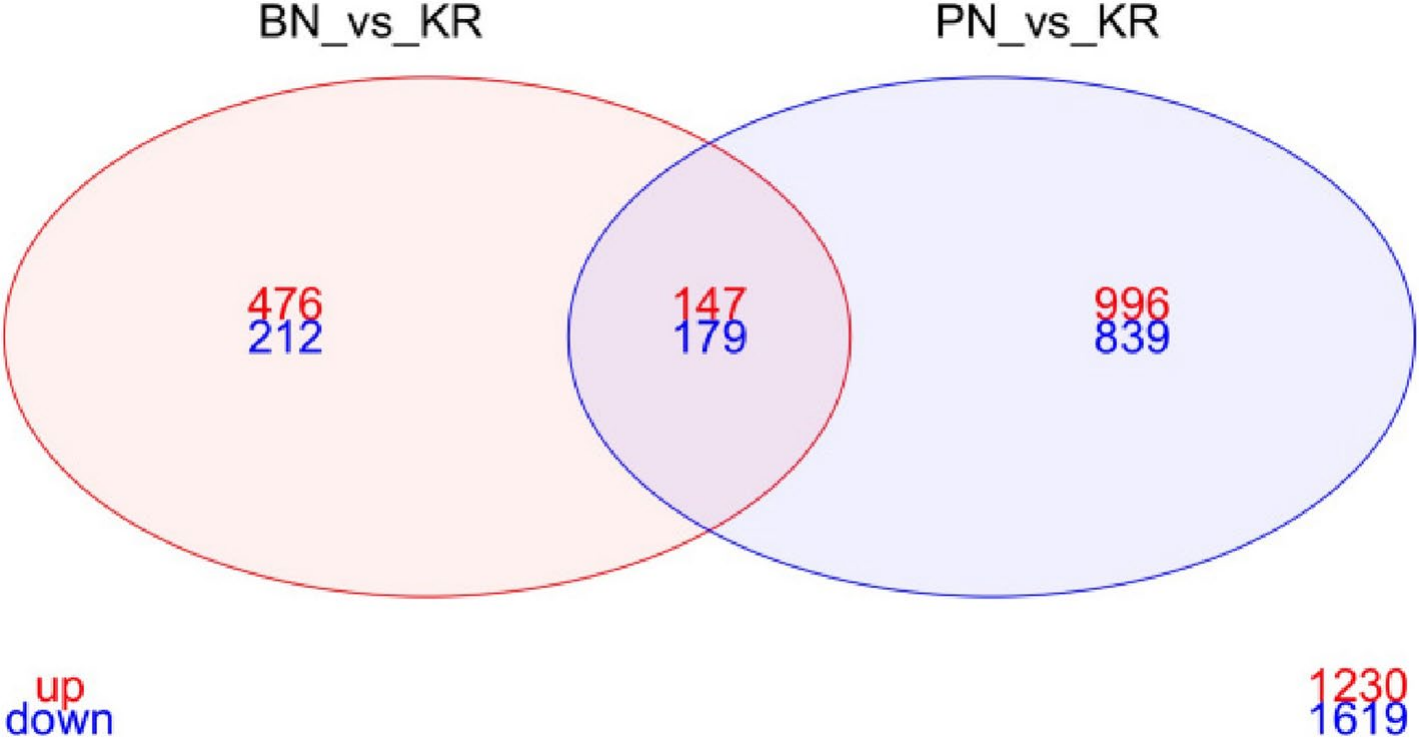
Venn diagram summarizing the numbers of differentially expressed (DE) genes between non-responders (N) and responders (R) with a corrected/adjusted *p*-value < 0.05 in the Bungoma and Pimperena populations

A total of 326 (147 upregulated and 179 down-regulated) DE genes were shared in the BN-KR vs PN-KR comparison (Fig. 4). Of the top shared genes, AGAP012916 (FC = 9.412813285) linked to mitochondrial respiratory chain complex II assembly was the most over expressed. Other DE gene terms included AGAP007293 and AGAP007292, both associated with negative regulation of apoptotic process; AGAP004157, linked to membrane activity; and AGAP012193, associated with NEDD8 transferase activity.

**Fig. 5:**
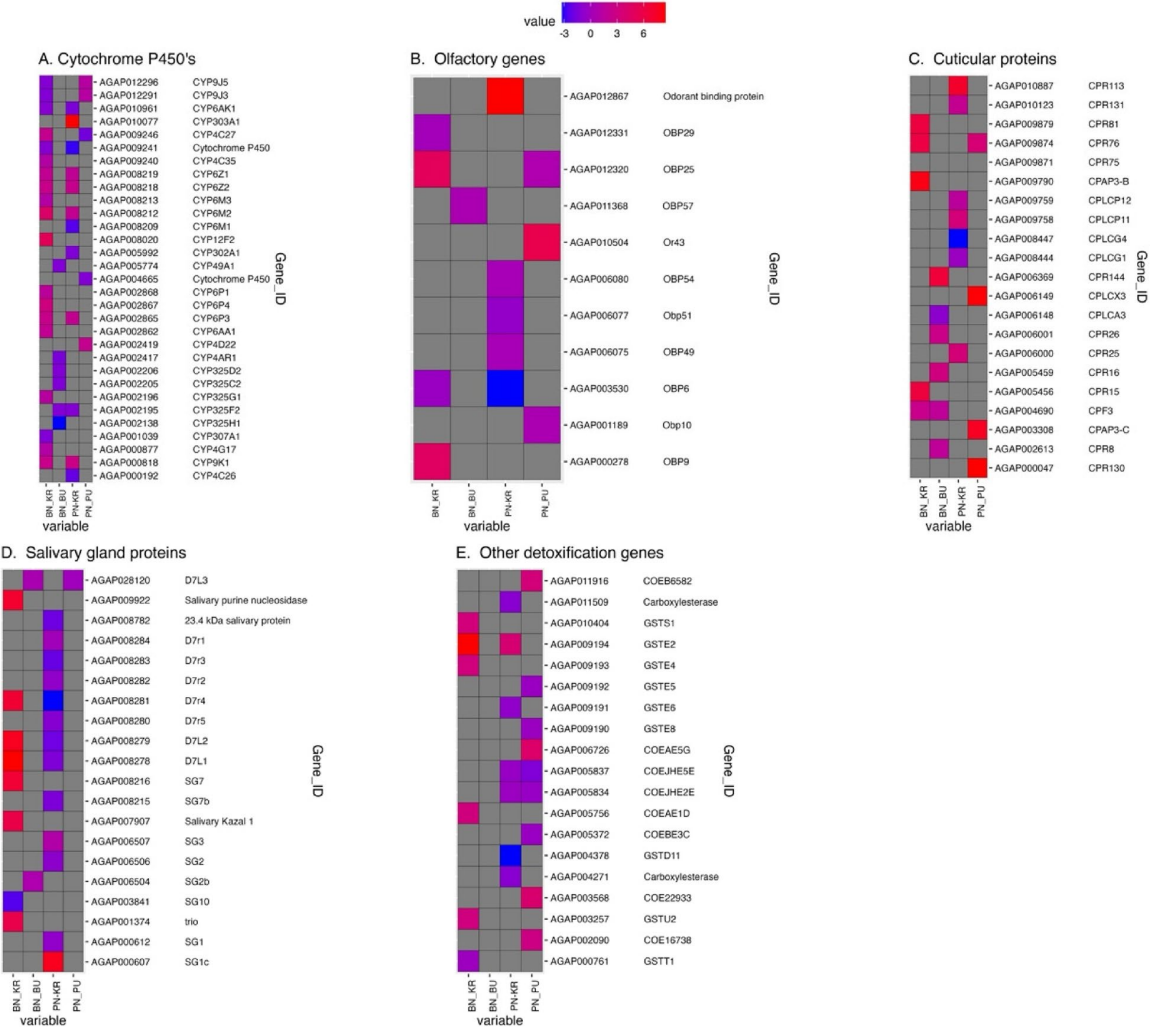
Heatmaps summarizing differentially expressed genes, showing log2 fold-change and *p* < 0.05 values on a red-blue scale (red = overexpressed; blue = down expressed). **A** Cytochrome P450 monooxygenases family. **B** Olfactory genes. **C** Cuticular proteins. **D** Salivary gland proteins. **E** Glutathione-S-transferases and carboxylesterases

Among the detoxification genes, CYP12 F2 was the most overexpressed cytochrome P450 gene (AGAP008020, FC = 36.6389 in Bungoma non-responder population); and one glutathione-s-transferase gene, GSTE2 (AGAP009184, FC = 28.3665 in Bungoma and FC = 3.07216 in Pimperena comparison) as summarized in the heatmaps in Fig. 5.

### Functional enrichment analysis of *An. gambiae s.s.* differentially expressed genes

A significant number of gene ontologies were enriched in the Bungoma transfluthrin non-responder mosquitoes, relative to the Kisumu responders. Of the top 10 most enriched terms, four terms (GO:0010951, GO:0010466, GO:0052548 and GO:0052547) were linked to negative regulation of endopeptidase activity. Two terms (GO:0022900 and GO:0022904) were associated with regulation of electron transport chain activity. GO:0006091 linked to generation of precursor metabolites and energy, GO:0045861 was associated with negative regulation of proteolysis, GO:0051346 linked to negative regulation of hydrolase activity and GO:0006693 was associated with prostaglandin metabolic process and were significantly enriched after *p*-value correction for multiple testing using the Benjamini–Hochberg method. Similarly, enriched gene sets were overexpressed in Pimperena transfluthrin non-responders relative to Kisumu responder mosquito populations. Of the top 10 most enriched terms, GO:0050794 was associated with regulation of cellular process; GO:0065007 and GO:0050789 linked to regulation of biological process; and GO:0007267, GO:0023052, GO:0099536, GO:0099537, GO:0007268 and GO:0098916 were involved in cell–cell and trans-synaptic signaling. Another, GO:0006468, linked to protein phosphorylation. All the enriched GO terms were then summarized using GO Figure! [30] as shown in Supplementary Material 6.

### Non-synonymous single nucleotide polymorphism (SNP) analysis

Two amino acid substitutions at codon 995 of the *vgsc* gene, associated with pyrethroid resistance, TCA (Serine) and TTT (Phenylalanine), were detected at high frequencies in the Bungoma and Pimperena populations, respectively. In the unexposed control, responder and non-responder Pimperena samples, a complete fixation of the *kdr*-L995 F mutation was observed. In the Bungoma population, the *kdr*-L995S allele was observed at a frequency of 100% in both non-responder and unexposed control groups, and 83% in the responder group (Table 3). The *Ace-1* mutation was observed in the Pimperena population, but it was not detected in the Kisumu and Bungoma populations. The N1570Y mutation, also known as the"super-*kdr*"mutation, shown to intensify the effect of L995 F-mediated pyrethroid resistance was detected in the Pimperena population. A novel M490I allele whose role is not known was observed to be fixed in the Kisumu susceptible population.

**Table 3:**
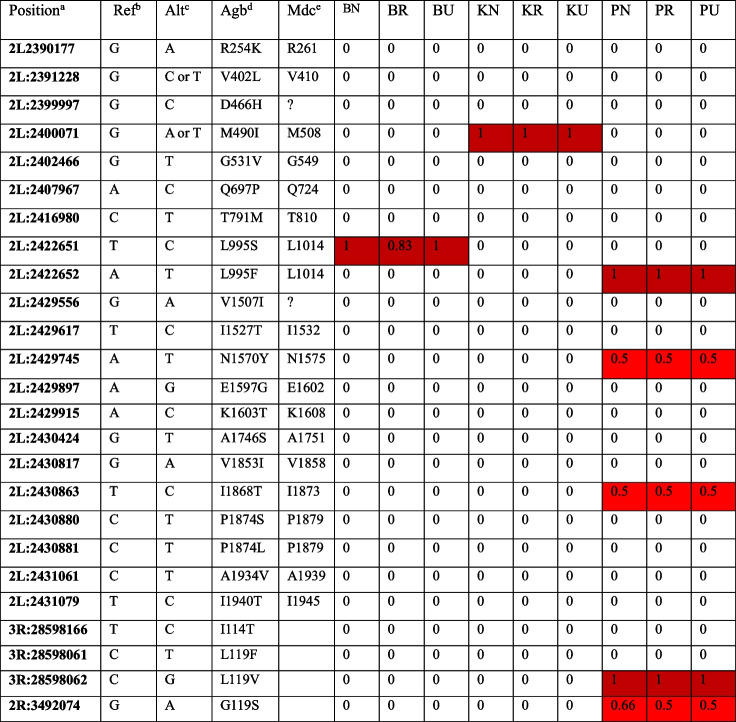
Summary of non-synonymous SNP detection analysis within the voltage-gated sodium channel and acetylcholineesterase-1 genes

^a^Position relative to the AgambP4 reference sequence, chromosome arm 2L

^b^Reference allele

^c^Alternative allele

^d^Codon numbering according to Anopheles gambiae transcript AGAP004707-RA in gene set AgamP4.4

^e^Codon numbering according to Musca domestica EMBL accession X96668 [33]

## Discussion

This study investigated the transcriptomic profiles associated with transfluthrin behavioural responses in *An. gambiae s.s.* mosquitoes. The behavioural spatial activity index (SAI) assays revealed heterogeneous responses to transfluthrin across the mosquito populations tested. The pyrethroid-resistant Pimperena strain required a much higher dose (125.15 μg/ml) to elicit a repellent response compared to the pyrethroid-susceptible Kisumu strain (0.0025 μg/ml). This suggests that behavioural resistance status impacts the efficacy of transfluthrin as a spatial repellent, consistent with previous findings in *Ae. aegypti* [19]. The field-collected Bungoma population showed an intermediate SAI (12.515 μg/ml), likely reflecting the heterogeneity of behavioural resistance in wild populations. The Bungoma *Anopheles gambiae s.s.* population has been previously demonstrated to be resistant to other pyrethroid class insecticides including permethrin [34], deltamethrin and alpacypermethrin [35, 36]. These results highlight the importance of monitoring the behavioural response to transfluthrin as part of routine resistance monitoring when implementing spatial repellent interventions.

The whole transcriptome analysis revealed several detoxification genes that were significantly overexpressed in the non-responders from resistant populations compared to the responders from susceptible strain. CYP12F2 showed very high fold changes in the Bungoma population (FC = 36.6389). This cytochrome P450 has previously been associated with pyrethroid resistance in *An. gambiae* from Cameroon [37]. Other upregulated detoxification genes included CYP6M2, CYP6Z3, CPAP3-B, CYP6P4 and GSTE2, which have been implicated in resistance to various insecticides in *An. gambiae s.l.* [38, 39]. The involvement of these genes suggests that specific metabolic resistance mechanisms may play a role in reducing behavioural responses to transfluthrin, despite previous assumptions that its unique chemical structure would limit P450-mediated detoxification [40]. Understanding how mechanisms that lead to increased detoxication of contact insecticides can also influence behavioural responses to volatile insecticides such as transfluthrin will have important implications for vector control strategies.

Previous studies have demonstrated that transfluthrin susceptibility is not highly enhanced when combined with P450 inhibitors such as piperonyl butoxide (PBO), 1-aminobenzotriazole, and triflumizole [17]. Fluorination of the transfluthrin benzyl ring is postulated to offer protection against possible detoxification by P450 s [40] and possibly the apparent lack of the 3-phenoxybenzyl moiety makes it less susceptible to P450 metabolism as previously illustrated in other pyrethroid-resistant insects like cotton bollworm [41]. Taken together, these contribute to the uncertainty around the specific mode of action of transfluthrin.

Further, transfluthrin has been shown to repel *Ae. aegypti* mosquitoes in hand-in-cage assays without detectable stimulation of antennal olfactory responses [42]. In this study, genes related to olfaction, such as odorant binding proteins and odorant receptors, were not significantly differentially expressed. This aligns with previous electrophysiological studies showing that transfluthrin does not stimulate olfactory receptor neurons at low concentrations [43]. Instead, the repellent effect may be mediated primarily through activation of voltage-gated sodium channels, as proposed by recent research [43].

A screening of non-synonymous single nucleotide polymorphisms in the voltage gate sodium (VGSC) gene at the L995 locus showed a correlation with high resistant allele frequency and behavioural non-response of *An. gambiae s.s.* mosquitoes from both the Bungoma and Pimperena populations. The single nucleotide polymorphism (SNP) analysis revealed fixation of the L995 F *kdr* mutation in the Pimperena population and high frequency of L995S in the Bungoma population. These mutations in the voltage-gated sodium channel gene have been widely associated with pyrethroid resistance in *An. gambiae s.l.* [31]*.* Their presence correlated with reduced transfluthrin sensitivity, suggesting that target-site resistance mechanisms may impact spatial repellency. Further, the N1570Y mutation was detected in the Pimperena population, and has been shown to intensify the effect of L995 F-mediated pyrethroid resistance. These findings have important implications for resistance management strategies and underscores the need for continued monitoring of *kdr* frequencies in malaria vector populations.

While these mutations indicate the presence of a codon and an approximate quantification, they cannot be translated into allele frequencies as the sequence reads were sampled from a pool of transcripts initially from a pool of 10 mosquitoes, having a potentially diverse contribution by each mosquito and thus allele. In order to determine allele frequencies more precisely, large pools of individual mosquitoes would be desirable as these could be used to give a much broader picture of point mutations segregating in each population. The BAM files were also parsed to screen for other known target-site mutations associated with resistance to non-pyrethroid insecticides. Target site mutations on the Acetylcholinesterase-1 (ACE-1) gene (target site for organophosphates and carbamates) that have been previously reported in *Anopheles gambiae s.s.* [31] were investigated. The G119S mutation was detected in the Pimperena population, but it was not detected in the Kisumu and Bungoma populations.

The fixation of the novel M490I allele in the otherwise susceptible Kisumu strain is intriguing. While its functional implications remain unknown, this mutation may represent a laboratory adaptation or genetic drift phenomenon in the long-maintained colony rather than a resistance-conferring mechanism, as evidenced by the strain’s-maintained susceptibility to pyrethroids. Further investigation of this mutation's functional significance is warranted. Gene ontology enrichment analysis highlighted several biological processes that may be involved in the mosquito behavioural responses to transfluthrin exposure. In the Bungoma population, terms related to serine-type endopeptidase activity were enriched, consistent with previous studies linking these enzymes to pyrethroid resistance [44]. For the Pimperena population, enriched terms included ion-gated channel activity, further supporting the role of voltage-gated sodium channels as probable mode of action and resistance to transfluthrin.

The Pimperena colony, with its high resistance to conventional insecticides, presented a unique opportunity to investigate the molecular mechanisms underpinning this phenotype [45]. Our initial hypothesis, based on the established insecticide resistance profile, predicted a minimal differential gene expression response. However, our screening revealed an unexpected pattern of divergence in gene expression between non-responders and unexposed individuals. This unexpected variability suggests the potential influence of unaccounted-for environmental factors within the screening chamber, besides the likely induction by transfluthrin treatment, further highlighting the need for a critical reassessment of our experimental paradigm in context of behavioural plasticity. To ensure the efficacy and reliability of future spatial repellent evaluations, it is imperative to invest in the development of novel bioassay methodologies or refine existing protocols that assess behavioral responses to volatile insecticides.

The significant overexpression of CYP12 F2 in transfluthrin non-responders represents a compelling candidate for further investigation. While our transcriptomic data strongly suggests its involvement in reduced behavioral sensitivity to transfluthrin, functional validation through techniques such as quantitative real-time PCR (qRT-PCR), in vitro expression systems, or RNAi-mediated gene silencing will be crucial in future subsequent studies. These approaches will help confirm the specific metabolic relationship between CYP12 F2 and transfluthrin, potentially revealing new targets for resistance monitoring and management.

## Conclusion

This study presents a comprehensive whole transcriptomic analysis of *An. gambiae s.s.* exhibiting differential behavioral responses to transfluthrin, offering significant new insights into the molecular mechanisms underlying this phenomenon. The observed overexpression of several detoxification genes in non-responders compared to responders, alongside the fixation of the L995 F *kdr* mutation in the Pimperena population (potentially enhanced by the N1570Y mutation) and the high frequency of L995S *kdr* mutation in the Bungoma population, strongly suggests their contribution to reduced transfluthrin behavioral sensitivity. Furthermore, the apparent upregulation of the cytochrome P450 monooxygenase gene family, particularly *CYP12 F2*, warrants further investigation as a potential factor in this insensitivity. Future functional validation of these candidate genes will be crucial for a more complete understanding of the molecular basis of behavioral resistance to transfluthrin.

## Supplementary Information

Supplementary Material 1. Descriptive statistics of RNA-Seq raw sequencing reads and alignment to Anopheles gambiae PEST reference genome.

Supplementary Material 2. A summary of the Principal Component Analysis (PCA) of RNASeq reads.

Supplementary Material 3. A hierarchical clustering heatmap of the Pearson’s correlation coefficient of the normalized expression profiles.

Supplementary Material 4. A detailed summary of all comparisons showing differentially expressed genes by population.

Supplementary Material 5. A summary of fold change values.

Supplementary Material 6. A summary of functional enrichment analysis of Anopheles gambiae ss.

## Data Availability

Raw RNA sequence data generated by this study is available at Sequence Read Archive (SRA) Bio Project accession number PRJNA986474. Custom scripts used for all the analyses are available from the authors on request.
