# Supplementary material for: Molecular markers of reduced behavioral sensitivity to transfluthrin in Anopheles gambiae s.s. from Western Kenya
Source: BMC Genomics. 2025 Jun 5;26:565. doi: 10.1186/s12864-025-11755-y (PMC12142849; doi:10.1186/s12864-025-11755-y)
Supplement: Supplementary file 1 — Supplementary Material 1. Descriptive statistics of RNA-Seq raw sequencing reads and alignment to Anopheles gambiae PEST reference genome. [file 12864_2025_11755_MOESM1_ESM.docx]

| Population |  | | | | | |
| --- | --- | --- | --- | --- | --- | --- |
|  | **Sample ID** | **Number of raw reads before** | **Number of reads after filtering** | **% of reads after filtering** | **Mapped Reads** | **% of mapped reads** |
|  |  |  |  |  |  |  |
| **Bungoma** | **BN01** | 53,775,562 | 51,957,652 | 97 | 47,829,069 | 92.05 |
|  | **BN02** | 92,142,452 | 89,419,362 | 97 | 83,040,981 | 92.87 |
|  | **BN03** | 62,986,480 | 61,168,276 | 97 | 56,810,606 | 92.88 |
|  | **BR01** | 85,089,820 | 82,839,118 | 97 | 76,904,990 | 92.84 |
|  | **BR02** | 30,622,252 | 29,989,298 | 98 | 24096399 | 80.35 |
|  | **BR03** | 33,121,712 | 32,251,548 | 97 | 28022678 | 86.89 |
|  | **BU01** | 23,947,920 | 23,216,248 | 97 | 21,737,985 | 93.63 |
|  | **BU02** | 69,739,516 | 67,896,680 | 97 | 62,867,948 | 92.59 |
|  | **BU03** | 114,315,628 | 111,345,070 | 97 | 102,526,410 | 92.08 |
| **Kisumu** | \| **KN01** \| \| --- \| | 76,204,572 | 74,371,548 | 98 | 70,901,869 | 95.33 |
|  | **KN03** | 72,286,212 | 67,890,950 | 94 | 66,351,506 | 97.73 |
|  | \| **KN02** \| \| --- \| | 74,729,728 | 72,847,024 | 97 | 68,165,040 | 93.57 |
|  | \| **KR01** \| \| --- \| | 63,613,472 | 61902604 | 97 | 57,868,337 | 93.48 |
|  | \| **KR02** \| \| --- \| | 74,412,060 | 72,296,668 | 97 | 69436286 | 96.04 |
|  | **KR03** | 54,771,474 | 53,295,586 | 97 | 51,470,628 | 96.58 |
|  | **KU03** | 53142 | 51384 | 97 | 41,849 | 81.44 |
|  | **KU01** | 71,183,974 | 68,611,890 | 96 | 67,284,123 | 98.06 |
|  | **KU02** | 47,294,674 | 45,755,548 | 97 | 44,237,918 | 96.68 |
| **Pimperena** | **PN01** | 59,728,028 | 57,828,518 | 97 | 52,521,022 | 90.82 |
|  | **PN02** | 75,042,824 | 72,383,266 | 96 | 65,197,650 | 90.07 |
|  | **PN03** | 58,162,084 | 55,348,808 | 95 | 49,042,940 | 88.61 |
|  | **PR01** | 54500144 | 51631350 | 94 | 34591099 | 67.00 |
|  | **PR02** | 72,269,344 | 69,615,028 | 96 | 60,865,676 | 87.43 |
|  | **PR03** | 70,115,074 | 68,025,596 | 97 | 63490627 | 93.33 |
|  | **PU01** | 93,958,128 | 90,373,264 | 96 | 83,122,438 | 91.98 |
|  | **PU02** | 77,700,466 | 75,339,088 | 97 | 71,978,372 | 95.54 |
|  | **PU03** | 68,931,004 | 65,875,206 | 96 | 59,020,974 | 89.60 |
