# Supplementary figures and images for: Molecular markers of reduced behavioral sensitivity to transfluthrin in Anopheles gambiae s.s. from Western Kenya
Source: BMC Genomics. 2025 Jun 5;26:565. doi: 10.1186/s12864-025-11755-y (PMC12142849; doi:10.1186/s12864-025-11755-y)

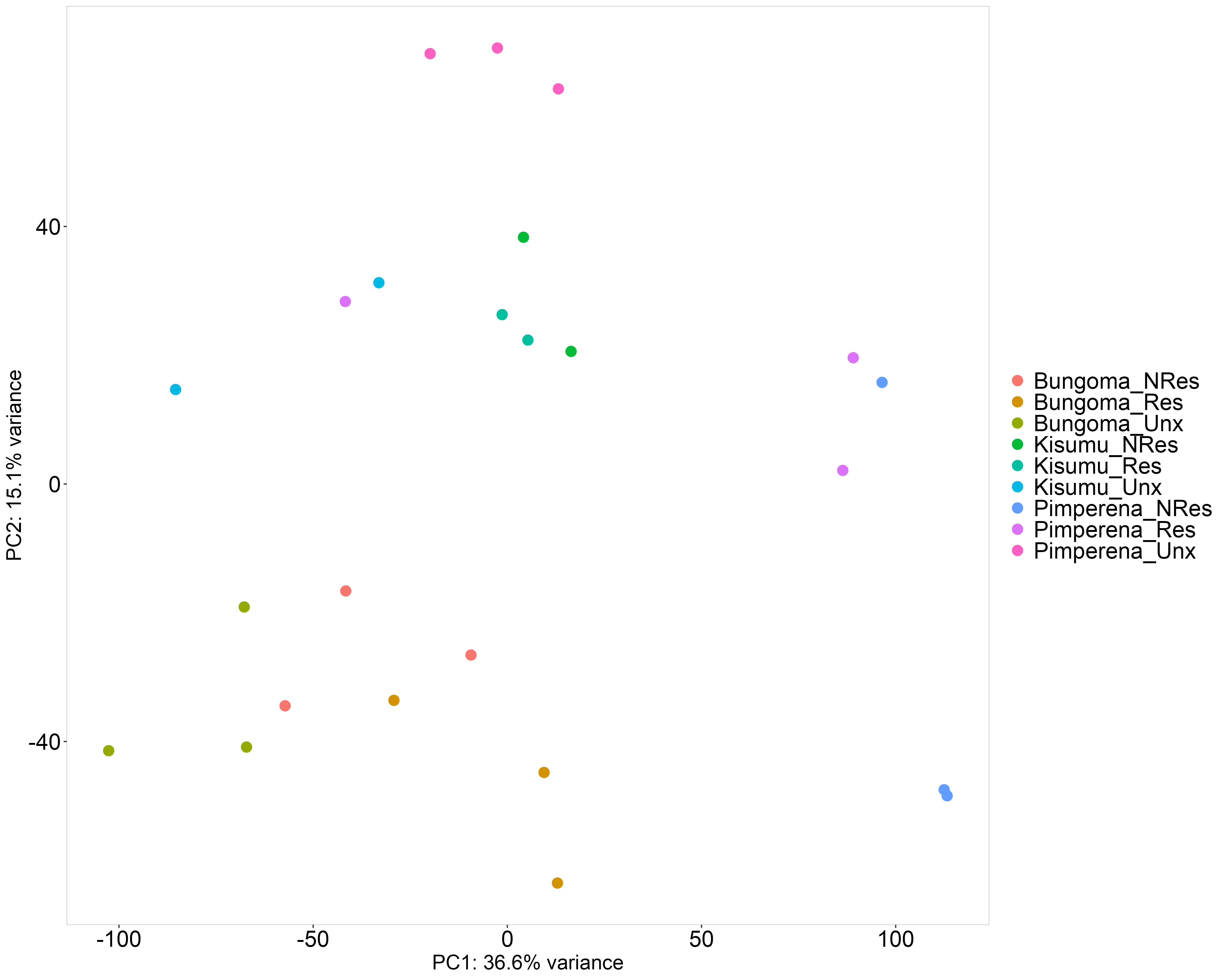

Supplement: Supplementary file 2 — Supplementary Material 2. A summary of the Principal Component Analysis (PCA) of RNASeq reads. [file 12864_2025_11755_MOESM2_ESM.png]

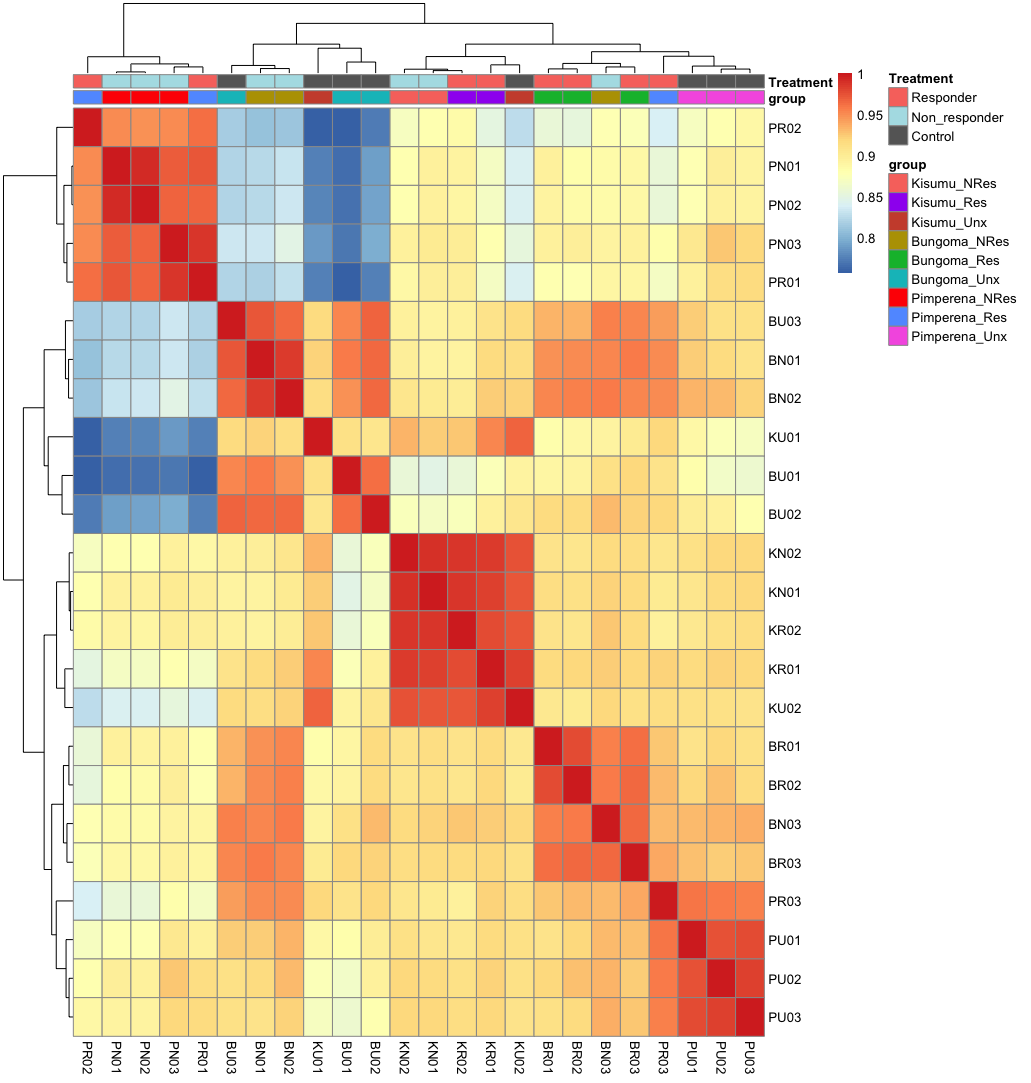

Supplement: Supplementary file 3 — Supplementary Material 3. A hierarchical clustering heatmap of the Pearson’s correlation coefficient of the normalized expression profiles. [file 12864_2025_11755_MOESM3_ESM.png]
