# Supplementary material for: Molecular markers of reduced behavioral sensitivity to transfluthrin in Anopheles gambiae s.s. from Western Kenya
Source: BMC Genomics. 2025 Jun 5;26:565. doi: 10.1186/s12864-025-11755-y (PMC12142849; doi:10.1186/s12864-025-11755-y)
Supplement: Supplementary file 4 — Supplementary Material 4. A detailed summary of all comparisons showing differentially expressed genes by population. [file 12864_2025_11755_MOESM4_ESM.docx]

| Comparison | No. of genes | DE genes(adjP<0.05) | | DE genes(adjP<0.01) | | DE genes(adjP<0.05) & (\|FC\| >2) | | DE genes(adjP<0.01) & (\|FC\| >2) | |
| --- | --- | --- | --- | --- | --- | --- | --- | --- | --- |
|  |  | **Up** | **Down** | **Up** | **Down** | **Up** | **Down** | **Up** | **Down** |
| PN vs KN | 9479 | 2377 | 2252 | 1877 | 1657 | 1100 | 997 | 1068 | 945 |
| PN vs KR | 9470 | 2384 | 2205 | 1886 | 1600 | 1160 | 1044 | 1111 | 981 |
| PN vs PU | 8943 | 2682 | 2729 | 2240 | 2017 | 1380 | 939 | 1351 | 897 |
| PU vs KN | 9431 | 1817 | 1858 | 1308 | 1355 | 642 | 730 | 631 | 697 |
| PU vs KR | 9406 | 1596 | 1711 | 1110 | 1223 | 572 | 713 | 550 | 652 |
| PN vs PR | 9528 | 0 | 1 | 0 | 1 | 0 | 1 | 0 | 1 |
| PN vs BR | 9656 | 2124 | 2304 | 1593 | 1653 | 962 | 1272 | 912 | 1158 |
| PN vs BN | 9711 | 2672 | 2746 | 2149 | 2089 | 1432 | 1533 | 1383 | 1471 |
| BN vs KN | 9768 | 1388 | 1006 | 971 | 594 | 863 | 450 | 797 | 408 |
| BN vs KR | 9748 | 741 | 981 | 637 | 432 | 649 | 408 | 565 | 342 |
| BN vs BU | 9996 | 205 | 263 | 99 | 142 | 214 | 111 | 78 | 136 |
| BU vs KN | 10054 | 2066 | 1792 | 1502 | 1185 | 1058 | 919 | 1304 | 837 |
| BU vs KR | 10047 | 1711 | 1486 | 1156 | 918 | 1191 | 795 | 1006 | 663 |
| BN vs BR | 9780 | 283 | 60 | 185 | 18 | 193 | 31 | 159 | 14 |
| BN vs PR | 9219 | 370 | 326 | 235 | 128 | 367 | 317 | 285 | 128 |

The table above shows a summary of results of differential gene expression analyses. DE = differentially expressed, FC = Fold change and adjP = P-value adjusted for multiple testing by the method of Benjamini and Hochberg (Benjamini & Hochberg, 1995).
