# Supplementary material for: Molecular markers of reduced behavioral sensitivity to transfluthrin in Anopheles gambiae s.s. from Western Kenya
Source: BMC Genomics. 2025 Jun 5;26:565. doi: 10.1186/s12864-025-11755-y (PMC12142849; doi:10.1186/s12864-025-11755-y)

**Summary visualizations of Gene Ontology Enrichment terms with GO Figure!**

BN_KR_down

Molecular_function

Molecular_function

Cellular components

Biological processes


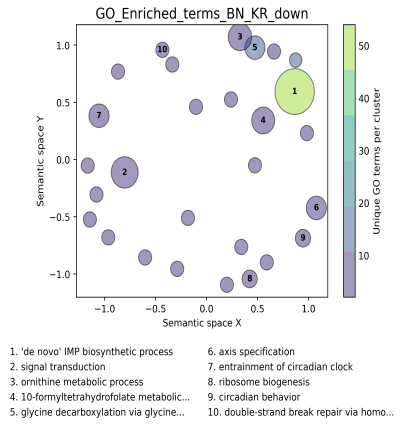

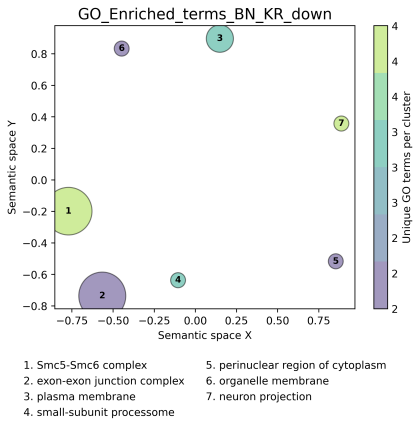

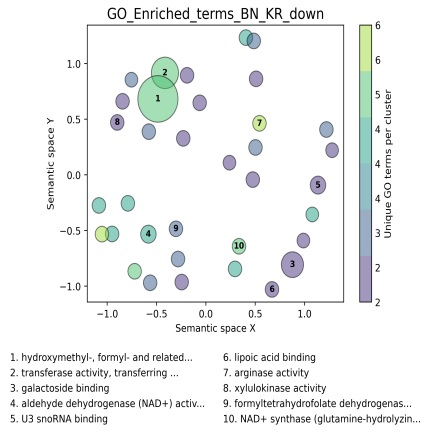


BN_KR_up

Molecular_function

Cellular components

Biological processes


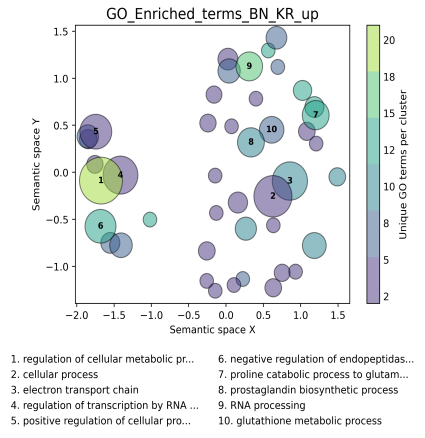

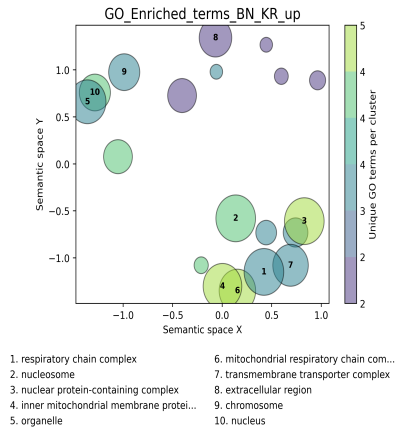

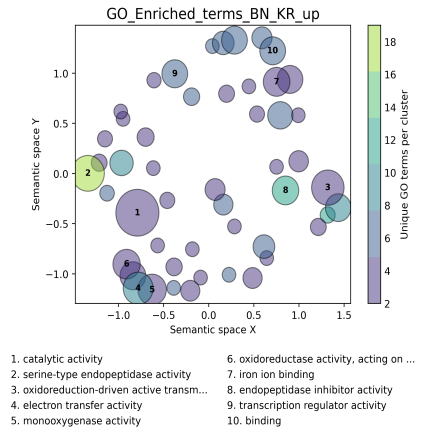


BN_BU_up

Biological processes

Biological processes

Cellular components

Molecular_function


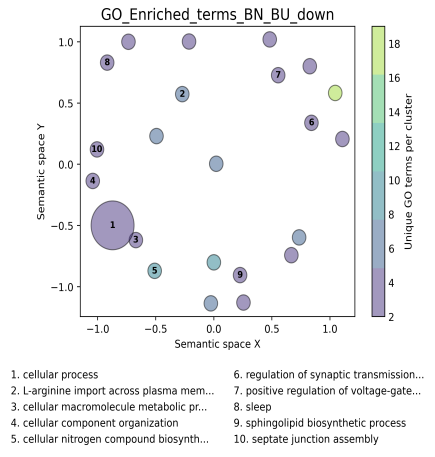

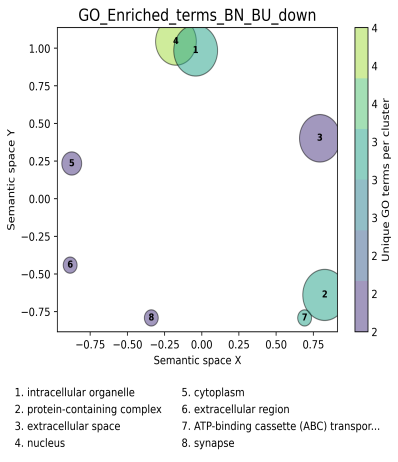

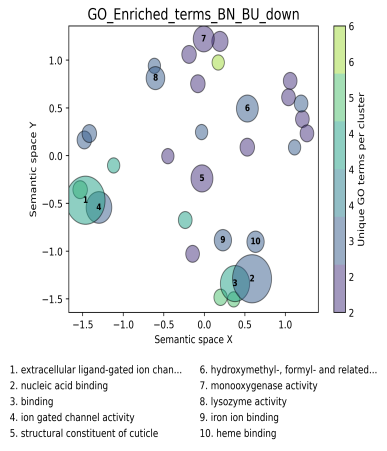


BU_KR_up


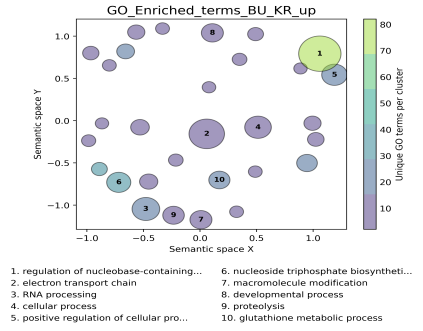

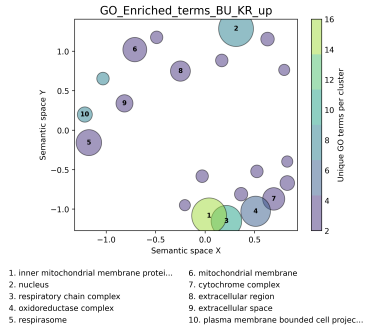

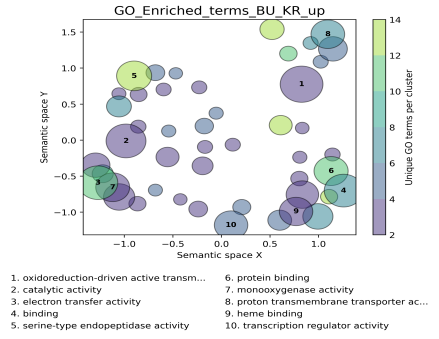


PN_KR_down

Biological processes


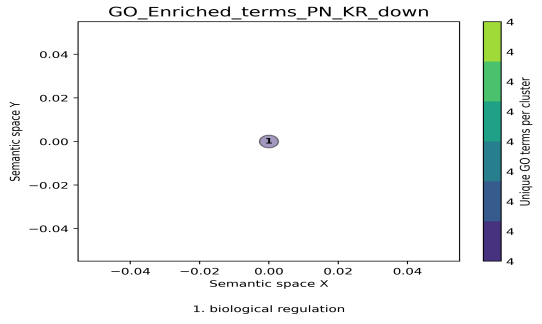


PN_KR_down


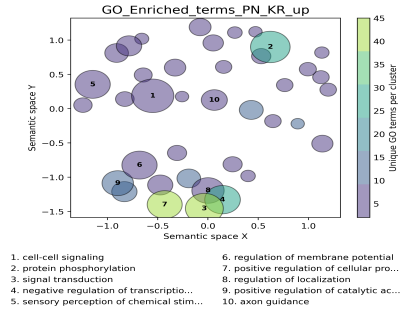

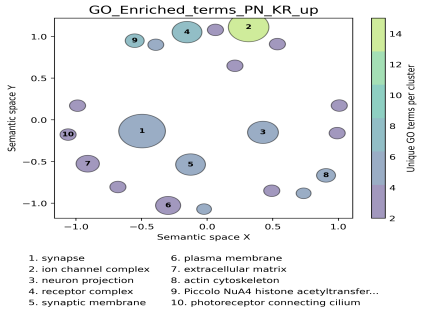

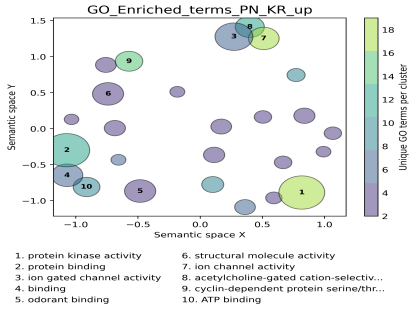


PN_PU_down


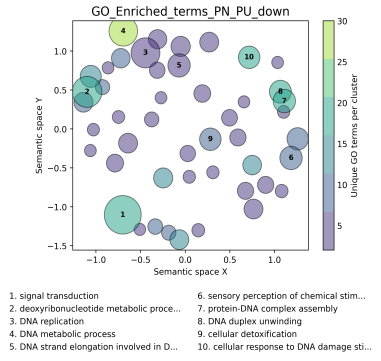

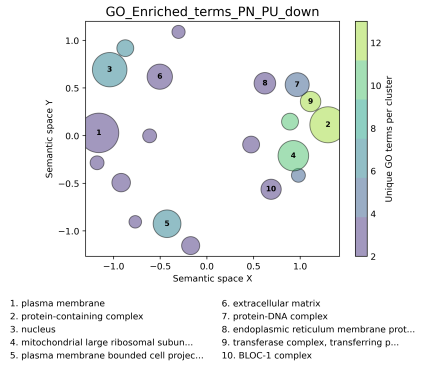

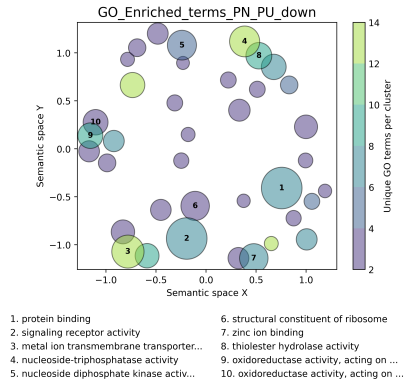


PN_PU_up


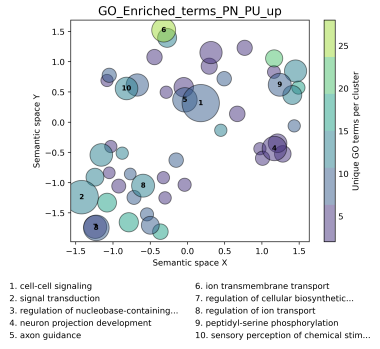

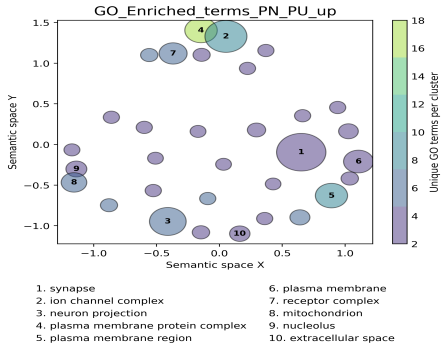

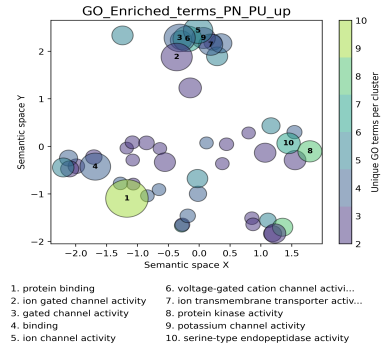


PU_KR_down


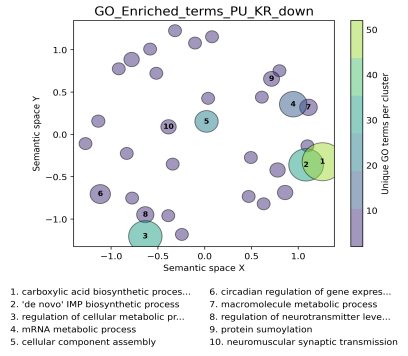

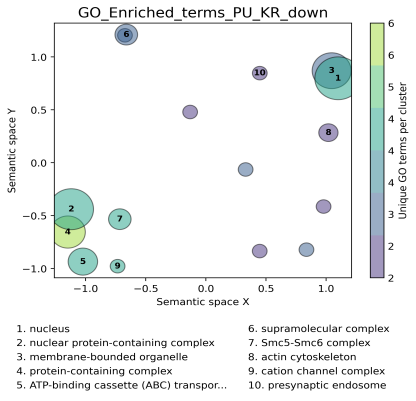

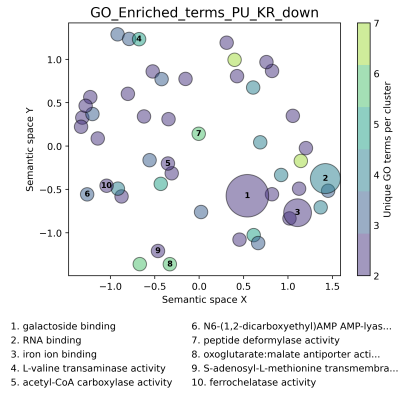


PU_KR_up


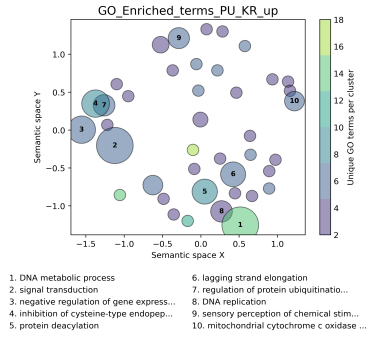

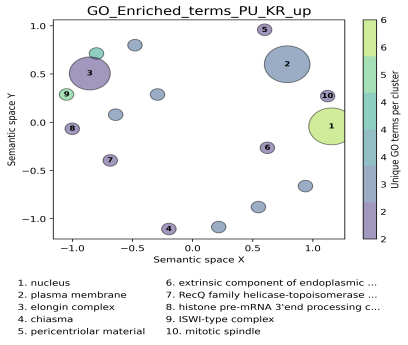

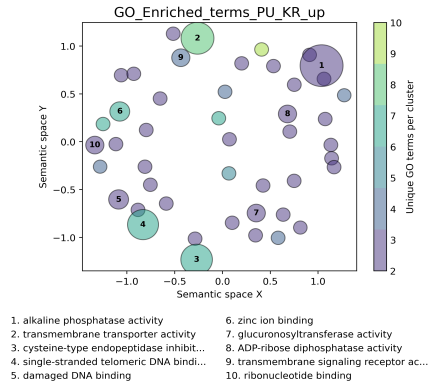

Supplement: Supplementary file 6 — Supplementary Material 6. A summary of functional enrichment analysis of Anopheles gambiae ss. [file 12864_2025_11755_MOESM6_ESM.docx]
